# Occurrence and Molecular Characterization of Extended-Spectrum Beta-Lactamase (ESBL)-Producing *Escherichia coli* in Broilers in Indonesia

**DOI:** 10.3390/antibiotics14101030

**Published:** 2025-10-15

**Authors:** Nur Hidayatullah, Imron Suandy, Montira Intanon, Thomas Alter, Oli Susanti, Ajeng Herpianti, Sani Susanty, Riska Desitania, Nattakarn Awaiwanont

**Affiliations:** 1 Veterinary Public Health and Food Safety Centre for Asia Pacific (VPHCAP), Faculty of Veterinary Medicine, Chiang Mai University, Chiang Mai 50100, Thailand; nur.hidayatullah@kalbarprov.go.id (N.H.); montira.intanon@cmu.ac.th (M.I.); 2 Department of Plantation and Livestock of West Kalimantan Province, Ministry of Agriculture, Jalan M. Hambal No. 3, Pontianak 76121, West Kalimantan, Indonesia; 3 Center of Veterinary Denpasar, Ministry of Agriculture, Jalan Raya Sesetan No. 266, Denpasar Selatan, Denpasar 80223, Bali, Indonesia; imron.suandy@pertanian.go.id; 4 Research Center of Producing and Development of Products and Innovations for Animal Health and Production, Faculty of Veterinary Medicine, Chiang Mai University, Chiang Mai 50100, Thailand; 5 School of Veterinary Medicine, Institute of Food Safety and Food Hygiene, Freie Universität Berlin, 14163 Berlin, Germany; thomas.alter@fu-berlin.de; 6 National Laboratory of Veterinary Public Health, Ministry of Agriculture, Jalan Pemuda No. 29A, Tanah Sereal, Bogor 16161, Jawa Barat, Indonesia; olisusanti@pertanian.go.id (O.S.); ajengherpianti@pertanian.go.id (A.H.); sani@pertanian.go.id (S.S.); riskadesitania@pertanian.go.id (R.D.)

**Keywords:** ESBL, *Escherichia coli*, phylogenetic group, ESBL gene, antimicrobial resistance, broiler, Indonesia

## Abstract

Extended-spectrum β-lactamase-producing *Escherichia coli* (ESBL-*E. coli*) are widespread in the food chain, but nationwide surveillance in Indonesian broiler production is limited. This study investigated the occurrence, antimicrobial resistance, phylogenetic diversity, and molecular characteristics of ESBL-*E. coli* from broilers in Indonesia. A total of 2182 *E. coli* isolates from broiler cecal samples across three regions during the period 2018–2020 were analyzed. Antimicrobial susceptibility testing and ESBL phenotyping were performed following the CLSI guidelines. ESBL resistance genes and phylogenetic groups were detected using multiplex/quadruplex PCR. ESBL-*E. coli* (9.9%) was most frequently observed in the western (15.2%) region, followed by the central (8.0%) and eastern (7.2%) regions. A total of 85 resistance patterns were identified, with 98.5% exhibiting multidrug resistance. The *bla*_CTX-M_ gene was detected in 97.5% of isolates, predominantly *bla*_CTX-M-1_ (97.5%), while *bla*_CTX-M-9_ was found in 2.5%. The *bla*_TEM_ gene was present in 33.0% of ESBL isolates; however, *bla*_SHV_ and *bla*_OXA-1_ were absent. Phylogenetic group A predominated (42.0%), followed by E (22.5%), B1 (20.5%), F (10.5%), C (2.5%), and D (2.0%). This study demonstrates a significant occurrence of ESBL-*E. coli* in Indonesian broilers with regional variation and *bla*_CTX-M_ predominance. The high rate of multidrug resistance poses a serious public health concern, emphasizing the urgent need for antimicrobial stewardship and enhanced surveillance programs.

## 1. Introduction

Chicken meat is a primary source of animal protein globally [1]. In Indonesia, poultry plays a pivotal role in the national diet, supplying over 65% of the animal protein consumed by its population of 275 million. As of 2023, the nation’s approximately 310 broiler establishments produced about 4.12 million metric tons of chicken meat annually, a figure expected to rise with population growth and consumer demand. However, the intensive nature of Indonesian poultry farming has generated conditions favorable for the development of antimicrobial resistance (AMR), posing significant risks to both animal health and public health [2,3]. This high production is often associated with widespread antimicrobial use, such as for prophylaxis, treatment, and even unauthorized growth promoters. The use of antimicrobials in poultry has accelerated the emergence of resistant pathogens, and many of these antimicrobials are critically important for treating human infections. 

The primary pathogens requiring management in Indonesian broiler systems include *Escherichia coli*, *Salmonella*, *Clostridium perfringens*, and respiratory pathogens such as *Mycoplasma gallisepticum*. Of particular concern is extended-spectrum beta-lactamase-producing *Escherichia coli* (ESBL-*E. coli*), which can hydrolyze β-lactam antibiotics, leading to treatment failure. ESBL enzyme production is primarily encoded by three major gene families: *bla*_TEM_, *bla*_SHV_, and *bla*_CTX-M_, with CTX-M-type β-lactamases being the most prevalent worldwide [4,5]. These resistance genes are often carried on mobile genetic elements such as plasmids and transposons, facilitating their horizontal transfer between bacterial species and contributing to the rapid dissemination of antimicrobial resistance across different bacterial populations [6]. The *bla*_CTX-M_ gene family is further subdivided into groups, with the CTX-M-1 and CTX-M-9 groups being among the most commonly detected in both clinical and veterinary isolates globally [7,8].

Surveillance of these resistant pathogens in food-producing animals is essential for understanding transmission dynamics and informing antimicrobial stewardship programs [9,10,11]. The importance of this surveillance extends beyond veterinary medicine, as ESBL-*E. coli* from poultry can directly impact human health through foodborne transmission, environmental contamination, and occupational exposure among farm workers and slaughterhouse personnel [12,13,14]. ESBL-*E. coli* infections are linked to increased morbidity, mortality, and healthcare costs [15].

Spatial variations in ESBL-*E. coli* prevalence are significant worldwide. Studies in Southeast Asia have shown varying prevalence rates, while European studies demonstrate considerable regional differences [16,17]. While studies have reported the presence of ESBL-*E. coli* in Indonesian poultry, these investigations are geographically limited and lack comprehensive national coverage, resulting in gaps that impede informed policy development [18,19,20]. Therefore, the objective of this study was to conduct a comprehensive nationwide investigation into the prevalence and molecular characteristics of ESBL-*E. coli* in broiler chickens across Indonesia. The findings provide essential baseline data to inform the country’s antimicrobial resistance surveillance and public health strategies.

## 2. Results

### 2.1. Third-Generation Cephalosporin-Resistant E. coli and ESBL-E. coli

Overall, out of 2182 *E. coli* isolates, 584 (26.8%) isolates were resistant to cefotaxime and/or ceftazidime. The highest percentage was observed in the western region (32.9%), followed by the central (28.2%) and eastern (20.7%) regions. In the analysis of ESBL phenotypic confirmation, 2023 isolates could be included. Among this subset, 425 isolates (21.0%) were resistant to cefotaxime and/or ceftazidime. ESBL-positive status was confirmed in 9.9% of the isolates tested. The prevalence of ESBL-*E. coli* was significantly higher in the western region than in the central (*p* < 0.001) and eastern (*p* < 0.001) regions. In contrast, there was no statistically significant difference in prevalence between the central and eastern regions (*p* = 0.844) (Table 1).

### 2.2. Antimicrobial Resistance of ESBL-E. coli

ESBL-*E. coli* isolates showed high rates of resistance to most antimicrobials tested (Table 2). The highest resistance rate was observed against cefotaxime, followed by ampicillin, sulfamethoxazole, trimethoprim, and gentamicin. Similar resistance rates were observed for the two quinolones: ciprofloxacin and nalidixic acid. Conversely, tigecycline and meropenem exhibited the lowest resistance rates.

Antimicrobial susceptibility testing revealed that the majority of ESBL-*E. coli* isolates (98.5%, 197/200) were multidrug-resistant (MDR), defined as exhibiting resistance to at least three antimicrobial classes. The most common profile was resistance to six antimicrobial classes (31.0%, 62/200), although some isolates (3.0%, 6/200) demonstrated resistance to as many as nine classes (Table 3).

The analysis of 200 ESBL-*E. coli* isolates revealed extensive phenotypic diversity, with 85 distinct antimicrobial resistance patterns (Table S1). The most prevalent resistance patterns demonstrated complex multidrug resistance involving eight–nine antimicrobial classes. The three most frequent patterns accounted for 23% of all isolates: (1) SMX+TMP+CIP+NAL+AZI+TET+CTX+AMP+GEN (8.0%, *n* = 16), (2) SMX+TMP+CIP+NAL+AZI+CAZ+CTX+AMP+GEN (7.5%, *n* = 15), and (3) SMX+TMP+CIP+NAL+AZI+TET+CAZ+CTX+AMP+GEN (7.5%, *n* = 15). Isolates with the most common resistance pattern were distributed across the western (*n* = 7), eastern (*n* = 6), and central (*n* = 3) regions. Notably, one isolate (0.5%) exhibited an extensively antimicrobial-resistant phenotype, with resistance to 13 antimicrobial agents (SMX+TMP+CIP+NAL+MER+AZI+CHL+TET+TGC+CAZ+CTX+AMP+GEN).

### 2.3. Antimicrobial Resistance Level of ESBL-E. coli

The MIC_50_ and MIC_90_ values revealed high levels of resistance to most antibiotics tested (Table 4). Resistance to beta-lactams was extensive, with cefotaxime showing an MIC_50_ of 4 μg/mL and MIC_90_ of >4 μg/mL, while ceftazidime showed an MIC_50_ of >8 μg/mL and MIC_90_ of >8 μg/mL. These high MIC values confirmed that over 90% of ESBL-*E. coli* isolates were resistant to third-generation cephalosporins, strongly reflecting active ESBL production, capable of hydrolyzing these antibiotics. Similarly, quinolone and fluoroquinolone resistance were extremely high, with ciprofloxacin having an MIC_50_ of 8 μg/mL and an MIC_90_ > 8 μg/mL, while nalidixic acid had an MIC_50_ of 128 μg/mL and an MIC_90_ > 128 μg/mL. This pattern demonstrated nearly complete resistance among isolates to this critical class of antibiotics. Resistance to sulfonamides was pronounced, with sulfamethoxazole showing an MIC_50_ > 1024 μg/mL and MIC_90_ > 1024 μg/mL, while trimethoprim exhibited an MIC_50_ of 32 μg/mL and MIC_90_ > 32 μg/mL. Additionally, high-level ampicillin resistance was observed across all isolates, with MIC_50_ and MIC_90_ values of 64 μg/mL and >64 μg/mL, respectively. Emergent colistin resistance was observed, with an MIC_90_ of 8 μg/mL; nevertheless, the majority of ESBL-*E. coli* isolates (85.5%) remained within the susceptible range. Among the tested agents, only meropenem and tigecycline demonstrated consistent effectiveness, with MIC_90_ values below established breakpoints; however, their application in poultry is limited.

A statistical analysis revealed significant regional differences in the geometric mean MICs for tetracycline and chloramphenicol (Table 5). The geometric mean MIC for tetracycline was significantly higher in the eastern region compared to the western region (*p* = 0.048). Furthermore, resistance to chloramphenicol was significantly higher in the western region than in both the eastern (*p* = 0.009) and central (*p* = 0.014) regions. For the other antibiotics, no statistically significant differences in geometric mean MICs were found among the regions (*p* > 0.05).

### 2.4. ESBL Resistance Genes of ESBL-E. coli Isolates from Broilers

All 200 ESBL-*E. coli* isolates were analyzed with specific primers for the presence of specific ESBL resistance genes. The most prevalent gene was *bla*_CTX-M_, including *bla*_CTX-M-1_ and *bla*_CTX-M-9_, followed by *bla*_TEM_ (Table 6). *bla*_SHV_ and *bla*_OXA-1_ were not detected in any of the isolates. *bla_CTX-M_*, specifically *bla*_CTX-M-1_, was the most dominant ESBL resistance gene across all regions. There were no statistically significant differences in the percentages of ESBL resistance genes between regions (*p* > 0.05).

Five ESBL resistance gene patterns of ESBL-*E. coli* isolates were identified. The most frequently observed was single *bla*_CTX-M-1_ (67.0%, 134/200), followed by *bla*_CTX-M-1_ + *bla*_TEM_ (28.0%, 56/200), single *bla*_TEM_ (2.5%, 5/200), *bla*_CTX-M-9_ + *bla*_TEM_ (1.5%, 3/200), and *bla*_CTX-M-1_ + *bla*_CTX-M-9_ + *bla*_TEM_ (1.0%, 2/200).

### 2.5. Phylogenetic Group

A phylogenetic analysis detected all *E. coli* phylogroups except for group B2 (Table 7). The most common phylogenetic group was group A, followed by E, B1, F, C, and D. The eastern and central regions exhibited similar phylogenetic distributions, with group A being the most frequent, followed by groups E and B1. In the western region, group A was also the most prevalent phylogroup, followed by groups B1, F, and E. In addition, phylogroup D was detected only in isolates from the western region. Although group C was found in all regions, it was only in a very small fraction. The only statistically significant difference among regions was for phylogroup E, which was found in a significantly higher proportion in the eastern region compared to the western region (*p* = 0.0003).

The distribution of ESBL resistance genes varied by phylogenetic group. While the *bla*_CTX-M1_ gene was highly prevalent (>95.0%) across all phylogroups, *bla_TEM_* showed a more varied distribution, occurring most frequently in group A (51.5%), followed by groups B1 (21.2%), E (18.2%), F (6.1%), and C (3.0%). Detection of the *bla*_CTX-M-9_ gene was restricted exclusively to phylogroups A (60.0%), B1 (20.0%), and C (20.0%).

## 3. Discussion

This study provides the first and most geographically comprehensive surveillance of ESBL-*E. coli* in Indonesian broiler production, establishing a unique region-specific national baseline. This nationwide surveillance revealed a 9.9% ESBL-*E. coli* prevalence across Indonesian broiler production, with significant regional variation. These findings contrast with previous localized studies in Indonesia reporting higher rates: 28.8% in West Java cloacal swabs and 25.0% in Bogor City feces [18,19]. Additionally, the prevalence in our study was significantly lower than that reported in Germany. That study reported 56.9% ESBL-*E. coli* from 51 cecal samples collected at slaughterhouses. These differences suggest regional variation in antimicrobial use patterns [21]. The lower percentage of ESBL-*E. coli* observed in this study when compared to other studies may be attributed to differences in sample size, geographic locations, sampling periods, and laboratory techniques [7,11,22]. The present study included more than 2000 *E. coli* isolates from different regions of Indonesia to reflect the ESBL-*E. coli* situation at the national level, while most other studies included smaller sample sizes and fewer *E. coli* isolates. A larger sample size improves the accuracy and reliability of ESBL-*E. coli* prevalence estimates. A small sample may not reflect the diversity of farm types, regions, or practices. The WHO report emphasizes that robust surveillance, including adequate sample sizes, is essential for accurately detecting antimicrobial resistance trends and informing public health interventions [23]. While most of the previous studies usually concentrate on one location or region, our study uses a collection of ESBL-*E. coli* isolates across three key geographical regions to establish a robust and essential national baseline. Antimicrobial resistance is usually dynamic; thus, collecting samples from different timeframes would also lead to differences in observed prevalences [24,25,26]. For screening of ESBL-*E. coli*, a recent study recommends utilizing MacConkey agar supplemented with 1 mg/L cefotaxime to improve both the sensitivity and specificity of ESBL-*E. coli* detection [27]. Therefore, further studies are suggested that employ cefotaxime-supplemented agar.

The results of this study highlight significant geographical disparities in ESBL-*E. coli* prevalence across Indonesia, with the western region showing a significantly higher prevalence of ESBL-*E. coli* compared to the central and eastern regions. This variation is likely attributable to differences in broiler production characteristics and practices, such as antimicrobial usage, farming practices, and biosecurity measures. Indonesia’s broiler population is heavily concentrated in its western and central regions, with significantly lower numbers in the eastern region [28]. Western Indonesia’s higher prevalence aligns with its concentration of intensive commercial farms using high stocking densities and rapid flock turnover. Such conditions can compromise biosecurity and encourage antimicrobial use, creating selective pressure for resistant bacteria. The higher population density and industrial activity in the west may also contribute to environmental reservoirs of resistant strains, while the movement of personnel like veterinarians and technicians between farms can facilitate their spread [29]. In contrast, the central region’s lower prevalence may be linked to moderately intensive farming and better farmer attitudes toward antimicrobial usage [24]. The eastern region exhibited the lowest prevalence, a finding attributed to its smaller and less concentrated broiler population, which consists mainly of small-scale, independent farms [25]. Although biosecurity in these eastern farms may not be ideal, the low underlying incidence of ESBL-*E. coli* in both broilers and humans limits the overall opportunity for transmission [26]. Thus, the structure of the regional broiler industry is one of the main drivers of ESBL-*E. coli*. This suggests that control efforts must be tailored by region, for example, reducing antibiotic dependency in the west and proactively protecting the central and eastern regions’ low-risk environment.

The extremely high prevalence of multidrug resistance (98.5%) among ESBL-*E. coli* isolates represents one of the most concerning findings in this study. This extensive MDR prevalence indicates severe resistance selection pressure within Indonesian broiler systems. The diversity of resistance patterns, with 85 distinct profiles identified, suggests widespread circulation of multiple-resistance plasmids and mobile genetic elements rather than clonal expansion of single resistant strains. The predominant pattern involving nine antimicrobial classes (SMX+TMP+CIP+NAL+AZI+TET+CTX+AMP+GEN) demonstrates the complexity of co-resistance mechanisms operating in Indonesian poultry production. High resistance rates to critically important antimicrobials pose significant therapeutic challenges. Third-generation cephalosporin resistance (cefotaxime 98.5%, ceftazidime 51.1%) confirms active ESBL production, capable of hydrolyzing these antibiotics, while extensive quinolone resistance (ciprofloxacin 72.0%, nalidixic acid 71.5%) threatens treatment options for human infections. These rates substantially exceed those reported in European studies: ciprofloxacin resistance reached only 10% in the Netherlands and 38% in Spain compared to our 72% [30,31,32]. Studies demonstrate that *E. coli* from quinolone-treated broilers exhibit significantly higher resistance levels than untreated ones [33], with poultry isolates showing greater ciprofloxacin resistance than those from pigs or humans due to more intensive quinolone use in poultry [22,34].

The Indonesian context reveals concerning antimicrobial usage patterns. According to 2017 data, approximately 80% of broiler farmers employ antimicrobials prophylactically, potentially driving bacterial resistance increases [35]. Sulfadiazine + trimethoprim represents the most widely used combination in broiler chickens [36], which correlates with sulfonamide resistance ranking among the top five classes in this study (86.0% for sulfamethoxazole, 84.0% for trimethoprim). Additionally, 96.9% of farmers utilize unauthorized commercial feeds containing antimicrobial growth promoters (penicillin, kanamycin, erythromycin, and oxytetracycline), further contributing to resistance development [37].

Colistin resistance emergence (14.5%) presents particularly alarming implications given its status as a last-resort antibiotic for multidrug-resistant Gram-negative infections. Although most isolates (85.5%) remained susceptible, detecting resistance genes warrants immediate enhanced surveillance. Among available treatment options, carbapenems such as meropenem remain the most reliable for ESBL-*E. coli* infections, while tigecycline demonstrates beneficial in vitro activity but its use should be limited to scenarios when carbapenems are unsuitable [38,39]. Minimal resistance to meropenem (3.0%) and tigecycline (2.5%) provides limited therapeutic alternatives, though their application in poultry remains restricted.

A minimum inhibitory concentration analysis revealed marked reductions in antimicrobial susceptibility, demonstrated by extremely elevated MIC_50_ and MIC_90_ values across important antimicrobial classes, including sulfonamides, quinolones, cephalosporins, penicillin, and aminoglycosides. The absence of significant regional differences in the geometric mean MICs for most antimicrobials tested suggests that nationwide resistance patterns are driven by common selective pressures. However, regional variation was observed for certain agents: tetracycline resistance was significantly higher in the east than in the west, whereas chloramphenicol resistance was more pronounced in the west compared to central and eastern regions. These findings imply that local factors, such as regional variation in antibiotic usage and the potential clonal spread of resistant strains, may underlie the distinct resistance profiles observed.

High co-resistance between fluoroquinolones (ciprofloxacin, nalidixic acid) and folate pathway inhibitors (sulfamethoxazole, trimethoprim) suggests clustered resistance genes [40,41]. These highly resistant strains in broiler production highlight the food chain as a significant vehicle for disseminating clinically important resistance to the public. Antimicrobial-resistant bacteria can spread through environmental contamination [42]. Enhanced biosecurity measures in livestock operations represent the most effective prevention strategy against multidrug-resistant bacterial contamination.

The molecular analysis revealed similar patterns in ESBL gene distribution across Indonesian broiler production. While *bla*_CTX-M_ genes dominated in all regions, the eastern region showed universal prevalence (100%), while the central (94.2%), and western (97.8%) regions demonstrated slightly lower prevalences. This regional uniformity differs from previous Indonesian findings, where *bla*_CTX-M_ was detected in only 6.0% of West Java samples, suggesting our larger sample size captured broader genetic diversity [43]. International comparisons reveal higher detection rates than the United Kingdom (15.6%), indicating Indonesia’s more widespread *bla*_CTX-M_ dissemination [35]. The predominance of *bla*_CTX-M-1_ variants (97.5%) over *bla*_CTX-M-9_ (2.5%) reflects global trends, where CTX-M-1 group enzymes have become increasingly prevalent. This shift from historical patterns where *bla*_TEM_ and *bla*_SHV_ predominated suggests that third-generation cephalosporin usage in Indonesian poultry has driven selective pressure favoring CTX-M-1 evolution. The absence of *bla*_SHV_ and *bla*_OXA-1_ genes distinguishes Indonesian broiler isolates from global patterns, where these genes commonly co-occur with *bla*_CTX-M_ variants.

The regional variation in *bla*_TEM_ prevalence (central: 38.5% vs. west: 27.0%) indicates different co-resistance patterns. As one of the earliest ESBL determinants described, *bla*_TEM_ continues to circulate widely in Enterobacteriaceae, often co-occurring with newer ESBL genes [44]. The co-existence of *bla*_TEM_ and *bla*_CTX-M_ on conjugative plasmids enhances multidrug resistance phenotypes and facilitates rapid horizontal gene transfer, with higher *bla*_TEM_ prevalence in central regions potentially correlating with penicillin usage patterns that favor ampicillin resistance mechanisms [26,45].

This molecular epidemiology pattern suggests established horizontal gene transfer networks across Indonesian broiler production, with conjugative plasmids, integrons, and transposons facilitating ESBL gene dissemination [46,47,48]. This combination of *bla*_CTX-M-1_ dominance with persistent *bla*_TEM_ circulation indicates that Indonesian ESBL evolution follows a distinct route, potentially reflecting specific antimicrobial selection pressures in local production systems. These findings underscore the importance of targeted molecular surveillance combined with antimicrobial usage monitoring to design effective stewardship interventions addressing the full spectrum of ESBL mechanisms in Indonesian poultry.

A phylogenetic analysis revealed diversity patterns, with important clinical implications. Group A’s predominance (42.0%) aligns with global broiler-associated studies, contrasting markedly with Brazilian livestock systems, where Group B1 dominated (57.0%) [49]. This difference suggests host-specific phylogenetic adaptation in Indonesian poultry systems. The detection of phylogroup F across all regions (east: 1.8%, central: 15.4%, west: 12.9%) represents a novel finding with significant public health implications. Unlike commensal groups A and B1, phylogroup D and F isolates possess enhanced virulence factors regardless of host origin, potentially facilitating zoonotic transmission and extraintestinal infections [50,51,52,53]. The higher prevalence in western regions correlates with increased production intensity, suggesting industrial farming practices may select for virulent lineages.

The detection of phylogroup D at low frequency in western regions (4.3%) demands immediate attention due to its correlation with significant human pathogenicity, suggesting potential environmental contamination sources that require improved biosecurity protocols in western production facilities. This molecular epidemiology pattern shows that Indonesian broiler production has developed a unique combination of resistance and virulence. This indicates that surveillance and intervention strategies need to be focused on both antimicrobial resistance and pathogenic potential.

This study has some limitations. First, the data, collected from 2018 to 2020, establish a critical national baseline but may not reflect current resistance dynamics. Second, our slaughterhouse-based sampling design precluded the collection of farm-level data, such as biosecurity, antimicrobial usage, and management practices, preventing the analysis of specific risk factors for regional variations. Finally, the use of whole-genome sequencing (WGS) would have offered more comprehensive insights into the resistance mechanisms. Therefore, we recommend that future surveillance studies incorporate longitudinal surveillance, on-farm data collection, and WGS to track resistance evolution and identify its drivers.

## 4. Materials and Methods

### 4.1. E. coli Isolation

**Figure 1:**
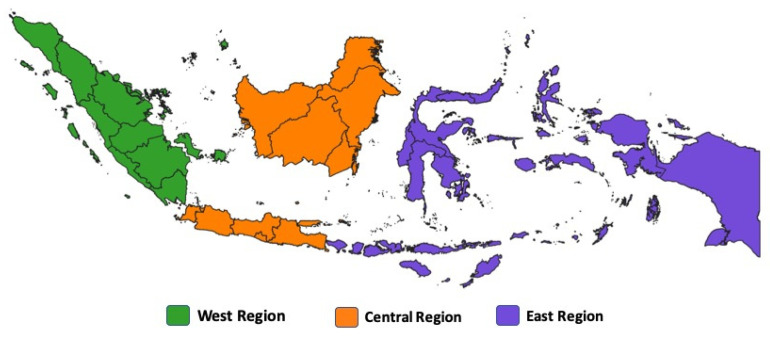
A map of the regions in Indonesia.

A total of 2182 *E. coli* isolates were obtained from the National Antimicrobial Resistance Surveillance Program in Indonesia. Briefly, broiler caecum samples were collected from slaughterhouses located across three major regions of Indonesia: West, central, and east, during the years 2018–2020 (Figure 1). Each cecum sample was randomly collected from one individual bird after the slaughtering procedure, thereby ensuring that each sample originated from a distinct farm source. The process of collecting samples was repeated every two weeks. *E. coli* isolation and identification were conducted in eight regional animal laboratories. Each individual cecal sample was streaked directly onto MacConkey agar and incubated at 37 °C for 18–24 h. Indole, methyl red, Voges–Proskauer, and citrate (IMViC) biochemical tests were conducted to verify the bacterial species. The *E. coli* colonies were cultivated on tryptic soy broth for 24 h and thereafter stored at a temperature of −20 °C in a supplement with 5% glycerol.

### 4.2. Antimicrobial Susceptibility Test

One phenotypically *E. coli* isolate per sample was sent to the National Quality Laboratory for Livestock Products, Bogor, Indonesia, and subjected to an antimicrobial susceptibility test using the Sensititre^TM^ Complete Automated AST System (Thermo Scientific^TM^, Waltham, MA, USA). A total of 14 types of antimicrobial agents among 10 antimicrobial classes were included: ampicillin (AMP), azithromycin (AZI), cefotaxime (CTX), ceftazidime (CAZ), chloramphenicol (CHL), ciprofloxacin (CIP), colistin (COL), gentamicin (GEN), meropenem (MER), nalidixic acid (NAL), sulfamethoxazole (SMX), tetracycline (TET), tigecycline (TGC), and trimethoprim (TMP). The results were interpreted according to the CLSI guidelines [54], with the exception of tigecycline, for which the EUCAST breakpoint was applied [55].

The isolates were classified as multidrug resistant (MDR) when resistant to at least one agent in three or more antimicrobial classes. The minimum inhibitory concentration (MIC) was recorded, and MIC_50_ and MIC_90_ were identified [56].

### 4.3. Phenotypic confirmation of ESBL-E. coli

*E. coli* isolates that showed resistance to third-generation cephalosporins (cefotaxime and/or ceftazidime) were evaluated for the production of the ESBL enzyme. According to the Clinical Laboratory Standards Institute’s (CLSI) standard, the double-disk diffusion test was used to phenotypically characterize ESBL-producing isolates [54]. In brief, cefotaxime (30 µg) and ceftazidime (30 µg), both alone and in combination with clavulanic acid (10 µg), were used. A bacterial cell suspension in 0.85% normal saline solution was made, and the turbidity was adjusted to match with a 0.5 McFarland standard. Subsequently, a sterile cotton swab was employed to put the suspension onto Mueller–Hinton agar (MHA) plates. Following the drying process, a cefotaxime (30 µg) antimicrobial disk and a clavulanic acid–cefotaxime (10 µg/30 µg) antimicrobial disk were placed on one cultured MHA plate. On an identical MHA plate, a ceftazidime (30 µg) antimicrobial disk and clavulanic acid–ceftazidime (10 µg/30 µg) antimicrobial disks were placed. The plates were incubated at 37 °C for 18–24 h [57]. The circular zones of inhibition around the antibiotic disks were measured according to the CLSI guidelines [54].

ESBL production was determined by a zone diameter > 5 mm for either antimicrobial agent when tested in combination with clavulanic acid compared to testing alone. *K. pneumoniae* ATCC 700603 and *E. coli* ATCC 25922 were used as quality control strains. Only ESBL-*E. coli* isolates were considered in the analysis of ESBL resistance genes, *E. coli* phylogenetic groups, and antimicrobial resistance patterns.

### 4.4. Determination of ESBL Resistance Genes

All of the ESBL-*E. coli* isolates were evaluated for the presence of ESBL resistance genes, *bla*_TEM_, *bla*_SHV_, *bla*_OXA-1_, *bla*_CTX-M-1_, *bla*_CTX-M-9_, and *bla*_CTX-M_, using the polymerase chain reaction (PCR) method. The genomic DNA of the *E. coli* isolate was extracted using a boiling method. Briefly, the *E. coli* isolates were cultured in 3 mL of Tryptone Soya Broth (Oxoid) supplemented with 1 mg/mL cefotaxime (Himedia) and incubated at 37 °C for 18–24 h. A volume of 1 mL of the culture was transferred to a 2 mL microcentrifuge tube, then centrifuged at 10,000× *g* for 5 min, and the supernatant was discarded. The pellet was suspended in 270 µL of nuclease-free water and heated at 99 °C for 10 min. The debris cell was discarded by centrifugation at 13,000× *g* for 2 min. The supernatant was collected. The genomic DNA was kept at −20 °C for further analysis.

Multiplex PCR was carried out to identify the *bla*_TEM_, *bla*_SHV_, *bla*_OXA-1_, *bla*_CTX-M-1_, and *bla*_CTX-M-9_ genes as previously described; all the primers used are listed in Table 8 [58,59]. The PCR reaction was performed under the following conditions: initial denaturation at 94 °C for 10 min, followed by 30 cycles of 30 s at 94 °C, 35 s at 61 °C, and 1 min at 72 °C, with a final 9 min extension at 72 °C. Amplicons (800 bp for *bla*_TEM_, 713 bp for *bla*_SHV_, 655 bp for *bla*_CTX-M-1_, 564 bp for *bla*_OXA-1_, and 518 bp for *bla*_CTX-M-9_) were visualized under UV light after electrophoresis through 2.5% agarose gel at 90 volts for 90 min.

The detection of *bla*_CTX-M_ genes was investigated using singleplex PCR as previously described [61]. The PCR reaction was performed under the following conditions: initial denaturation at 94 °C for 10 min, followed by 30 cycles of 30 s at 94 °C, 35 s at 60 °C, and 1 min at 72 °C, with a final 9 min extension at 72 °C. The amplicon (585 bp) was visualized under UV light after electrophoresis through 1.5% agarose gel at 100 volts for 30 min. Positive amplicons from each gene were purified using the gel extraction kit (PureDireX, Bio-Helix Co., Ltd., New Taipei city, Taiwan) and subjected to Sanger sequencing (ATGC, Co., Ltd., Pathum Thani, Thailand). The obtained sequences were evaluated using the Benchling version 2025.5 (Benchling Inc., San Francisco, CA, USA) and were compared with the reference sequences at GenBank.

### 4.5. Determination of E. coli Phylogenetic Group

The phylogenetic group (A, B1, B2, C, D, E, and F) was determined using a quadruplex phylogroup assignment method, as previously described by Clermont, Christenson et al. [62].

### 4.6. Statistical Analysis

To determine if the observed differences in percentages between the three regions were statistically significant, a chi-square test of independence was performed, and Tukey’s honestly significant difference (HSD) test was conducted as a post hoc analysis to identify the differences between each region. The MIC_50_, MIC_90_, MIC ranges, and geometric means of the MICs were calculated for each antibiotic, and a one-way analysis of variance (ANOVA) was performed to determine if there were statistically significant differences between regions; a more detailed statistical analysis using pairwise t-tests was conducted. The t-tests were performed on the log-transformed MIC values. Statistical analyses were performed by using the SAS statistical software (SAS. 2018. SAS University Edition: Statistics, 6th ed.), results were considered statistically significant if *p* < 0.05.

## 5. Conclusions

This study reveals a low occurrence of ESBL-*E. coli* in Indonesian broilers from 2018 to 2020, providing crucial nationwide data on its prevalence and regional gene distribution. These findings serve as a reference for government and stakeholders to develop strategies aimed at preventing the spread of antimicrobial resistance from poultry to humans and the environment. Future surveillance efforts should prioritize temporal trend analysis to monitor changes in ESBL-*E. coli* prevalence and evaluate the effectiveness of antimicrobial stewardship interventions. This should be complemented by comprehensive risk factor analysis to identify key transmission drivers. Furthermore, whole-genome sequencing should be employed to provide deeper insights into the molecular epidemiology, genetic relatedness, and transmission dynamics of these resistant strains across different regions and production systems.

## Figures and Tables

**Table 1:** Number and percentage of ESBL-*E. coli* in different regions.

Region	Cefotaxime and/or Ceftazidime-Resistant *E. coli*				ESBL-*E. coli*			
	N	Positive (%)	95% CI	*p*-Value for Regions Differences	N	Positive (%)	95% CI	*p*-Value for Regions Differences
East	808	167 (20.7) ^a^	18.1–23.6	<0.001	767	55 (7.2) ^a^	5.5–9.2	<0.001
Central	742	209 (28.2) ^b^	25.1–31.5		646	52 (8.0) ^a^	6.2–10.4	
West	632	208 (32.9) ^b^	29.4–36.7		610	93 (15.2) ^b^	12.6–18.3	
Total	2182	584 (26.8)			2023	200 (9.9)		

CI = confidence interval; within columns, percentages with different superscript letters indicate significant differences (*p*-value < 0.05).

**Table 2:** The rates of antimicrobial resistance of ESBL-*E. coli.*.

Antibiotic	Resistance Status (%)		
	R	I	S
Cefotaxime	98.5	0.0	1.5
Ampicillin	97.5	0.5	2.0
Sulfamethoxazole	86.0	0.0	14.0
Trimethoprim	84.0	0.0	16.0
Gentamicin	84.0	0.5	15.5
Ciprofloxacin	72.0	16.0	12.0
Nalidixic acid	71.5	0.0	28.5
Azithromycin	62.5	0.0	37.5
Tetracycline	55.0	1.5	43.5
Ceftazidime	51.1	34.0	15.0
Chloramphenicol	17.5	4.5	78.0
Colistin	14.5	85.5	0.0
Meropenem	3.0	0.5	96.5
Tigecycline	2.5	0.0	97.5

R = resistant, I = intermediate, S = susceptible.

**Table 3:** Percentages of ESBL-*E. coli* resistance to different numbers of antimicrobial classes.

No. of Antimicrobial Classes	No. of ESBL-*E. coli* Isolates	Percentage
2	3	1.5%
3	4	2.0%
4	16	8.0%
5	39	19.5%
6	62	31.0%
7	56	28.0%
8	14	7.0%
9	6	3.0%
Total	200	100%

**Table 4:** MIC_50_ (µg/mL) and MIC_90_ (µg/mL) values of ESBL-*E. coli* across Indonesia.

Antibiotic	Class	MIC_50_ (µg/mL)	MIC_90_ (µg/mL)	GM (µg/mL)	MIC Range	MIC Breakpoint (µg/mL)
Sulfamethoxazole	Sulfonamides	>1024	>1024	576.0	≤8–>1024	≥512
Trimethoprim	Sulfonamides	32	>32	15.6	≤0.25–>32	≥16
Ciprofloxacin	Quinolones	8	>8	2.5	0.03–>8	≥1
Nalidixic Acid	Quinolones	128	>128	56.7	≤4–>128	≥32
Colistin	Polymyxins	1	8	1.4	≤1–>16	≥4
Meropenem	Carbapenems	0.03	0.06	0.041	≤0.03–>16	≥4
Azithromycin	Macrolides	32	64	22.6	≤2–>64	≥32
Tetracycline	Tetracyclines	32	>64	13.4	≤2–>64	≥16
Chloramphenicol	Phenicols	8	128	12.5	≤8–>128	≥32
Tigecycline	Tetracyclines	0.25	0.5	0.282	≤0.25–4	>0.5
Ceftazidime	Cephalosporins	>8	>8	6.9	≤0.5–>8	≥16
Cefotaxime	Cephalosporins	4	>4	3.8	≤0.25–>4	≥4
Ampicillin	Penicillins	64	>64	59.3	1–>64	≥32
Gentamicin	Aminoglycosides	32	>32	18.3	≤0.5–>32	≥16

GM = geometric mean.

**Table 5:** MICs of antibiotics against ESBL-*E. coli* in broilers in different regions of Indonesia.

Antibiotic	Region	N	MIC_50_ (µg/mL)	MIC_90_ (µg/mL)	GM (µg/mL)	*p*-Value
Sulfamethoxazole	East	55	1024	1024	667.1	0.721
	Central	52	1024	1024	600.8	
	West	93	1024	1024	515.8	
Trimethoprim	East	55	32	32	17.9	0.712
	Central	52	32	32	15.6	
	West	93	32	32	14.3	
Ciprofloxacin	East	55	8	8	3.0	0.051
	Central	52	2	8	1.8	
	West	93	8	8	2.8	
Nalidixic Acid	East	55	128	128	51.7	0.270
	Central	52	128	128	48.4	
	West	93	128	128	65.4	
Colistin	East	55	1	1.6	1.3	0.614
	Central	52	1	3.8	1.3	
	West	93	1	8	1.6	
Meropenem	East	55	0.03	0.162	0.051	0.095
	Central	52	0.03	0.06	0.037	
	West	93	0.03	0.03	0.037	
Azithromycin	East	55	32	64	28.6	0.085
	Central	52	32	64	19.0	
	West	93	32	64	21.7	
Tetracycline	East	55	64	64	17.9 ^a^	0.044
	Central	52	64	64	15.8 ^ab^	
	West	93	8	64	10.3 ^b^	
Chloramphenicol	East	55	8	32	10.4 ^a^	0.036
	Central	52	8	15.2	10.4 ^a^	
	West	93	8	128	15.4 ^b^	
Tigecycline	East	55	0.25	0.5	0.302	0.629
	Central	52	0.25	0.475	0.271	
	West	93	0.25	0.25	0.277	
Ceftazidime	East	55	8	8	7.1	0.607
	Central	52	8	8	6.7	
	West	93	8	8	6.8	
Cefotaxime	East	55	4	4	3.4	0.064
	Central	52	4	4	4.0	
	West	93	4	4	3.9	
Ampicillin	East	55	64	64	56.4	0.528
	Central	52	64	64	59.1	
	West	93	64	64	61.2	
Gentamicin	East	55	32	32	16.4	0.570
	Central	52	32	32	19.8	
	West	93	32	32	18.7	

GM = geometric mean; different superscript letters indicate significant differences (*p*-value < 0.05).

**Table 6:** Distribution of ESBL resistance genes among ESBL-*E. coli* from broilers.

Region	N	ESBL Resistance Genes					
		*bla*_CTX-M_	*bla*_CTX-M-1_	*bla*_CTX-M-9_	*bla*_TEM_	*bla*_SHV_	*bla*_OXA-1_
East	55	55 (100%)	55 (100%)	1 (1.8%)	19 (34.5%)	0	0
Central	52	49 (94.2%)	49 (94.2%)	3 (5.8%)	20 (38.5%)	0	0
West	93	91 (97.8%)	91 (97.8%)	1 (1.1%)	27 (29.0%)	0	0
Total	200	195 (97.5%)	195 (97.5%)	5 (2.5%)	66 (33.0%)	0	0

**Table 7:** Phylogenetic group of ESBL-*E. coli* from broilers in different regions of Indonesia.

Region	Phylogenetic Group							Total
	A	B1	B2	C	D	E	F	
East	24 (43.6%) ^a^	7 (12.7%) ^a^	0 (0.0%) ^a^	2 (3.6%) ^a^	0 (0.0%) ^a^	21 (38.2%) ^a^	1 (1.8%) ^a^	55
Central	17 (32.7%) ^a^	12 (23.1%) ^a^	0 (0.0%) ^a^	1 (1.9%) ^a^	0 (0.0%) ^a^	14 (26.9%) ^a,b^	8 (15.4%) ^a^	52
West	43 (46.2%) ^a^	22 (23.7%) ^a^	0 (0.0%) ^a^	2 (2.2%) ^a^	4 (4.3%) ^a^	10 (10.8%) ^b^	12 (12.9%) ^a^	93
Total	84 (42.0%)	41 (20.5%)	0 (0.0%)	5 (2.5%)	4 (2.0%)	45 (22.5%)	21 (10.5%)	200

Within columns, percentages with different superscript letters indicate significant differences (*p*-value < 0.05).

**Table 8:** Primers used for detection of ESBL resistance genes.

Gene Targeted	Sequence (5′-3′)	Amplicon Size (bp)	Purpose	Reference
*bla*_TEM_	CATTTCCGTGTCGCCCTTATTC CGTTCATCCATAGTTGCCTGAC	800	Multiplex PCR	[60]
*bla*_SHV_	AGCCGCTTGAGCAAATTAAAC ATCCCGCAGATAAATCACCAC	713	Multiplex PCR	[60]
*bla*_OXA-1_	GGCACCAGATTCAACTTTCAAG GACCCCAAGTTTCCTGTAAGTG	564	Multiplex PCR	[60]
*bla*_CTX-M-1_	TTAGGAAGTGTGCCGCTGTA CGGTTTTATCCCCCACAAC	655	Multiplex PCR	[59]
*bla*_CTX-M-9_	GGTGATGAACGCTTTCCAAT TTATCACCTGCAGTCCACGA	518	Multiplex PCR	[59]
*bla*_CTX-M_	CGATGTGCAGTACCAGTAA TTAGTGACCAGAATCAGCGG	585	Singleplex PCR	[61]

## Data Availability

The original contributions presented in this study are included in the article/Supplementary Materials.

## Supplementary Materials

The following supporting information can be downloaded at: https://www.mdpi.com/article/10.3390/antibiotics14101030/s1, Table S1: Antimicrobial resistance patterns of ESBL-*E. coli.*

## References

[1] FAO (2024). Meat Market Review: Overview of Global Market Developments in 2023.

[2] Wahyono N., Utami M. (2018). A Review of the Poultry Meat Production Industry for Food Safety in Indonesia. J. Phys. Conf. Ser..

[3] DGLAHS (2018). Survey of Antimicrobial Usage in Poultry Farms in Indonesia.

[4] Ejaz H., Qamar M.U., Farhana A., Younas S., Batool A., Lone D., Atif M., Alruways M.W., Alruwaili M., Hamad I. (2024). The Rising Tide of Antibiotic Resistance: A Study on Extended-Spectrum Beta-Lactamase and Carbapenem-Resistant *Escherichia coli* and *Klebsiella pneumoniae*. J. Clin. Lab. Anal..

[5] Husna A., Rahman M.M., Badruzzaman A.T.M., Sikder M.H., Islam M.R., Rahman M.T., Alam J., Ashour H.M. (2023). Extended-Spectrum beta-Lactamases (ESBL): Challenges and Opportunities. Biomedicines.

[6] Zhang H.J., Wang H.W., Tian F.Y., Yang C.Z., Zhao M., Ding Y.X., Wang X.Y., Cui X.Y. (2024). Decolonization strategies for ESBL-producing or carbapenem-resistant Enterobacterales carriage: A systematic review and meta-analysis. Sci. Rep..

[7] Liu Y., Wang Y., Walsh T. (2016). Emergence of plasmid-mediated colistin resistance mechanism MCR-1 in animals and human beings in China: A microbiological and molecular biological study. Lancet Infect. Dis..

[8] Smith H.Z., Hollingshead C.M., Kendall B. (2025). Carbapenem-Resistant Enterobacterales.

[9] Tamma P.D., Heil E.L., Justo J.A., Mathers A.J., Satlin M.J., Bonomo R.A. (2024). Infectious Diseases Society of America 2024 Guidance on the Treatment of Antimicrobial-Resistant Gram-Negative Infections. Clin. Infect. Dis..

[10] Ribeiro L.F., Nespolo N.M., Rossi G.A.M., Fairbrother J.M. (2024). Exploring Extended-Spectrum Beta-Lactamase (ESBL)-Producing *Escherichia coli* in Food-Producing Animals and Animal-Derived Foods. Pathogens.

[11] Or P., Boonyayatra S., Punyapornwithaya V., Awaiwanont N. (2024). Prevalence of Extended-Spectrum Beta-Lactamase-Producing *Escherichia Coli* in Broiler Farms: A Systematic Review and Meta-Analysis. Vet. Integr. Sci..

[12] Mandujano-Hernandez A., Martinez-Vazquez A.V., Paz-Gonzalez A.D., Herrera-Mayorga V., Sanchez-Sanchez M., Lara-Ramirez E.E., Vazquez K., de Jesus de Luna-Santillana E., Bocanegra-Garcia V., Rivera G. (2024). The Global Rise of ESBL-Producing *Escherichia coli* in the Livestock Sector: A Five-Year Overview. Animals.

[13] Aliyu A.B., Jalila A., Saleha A.A., Zunita Z. (2024). ESBL Producing *E. coli* in Chickens and Poultry Farms Environment in Selangor, Malaysia: A Cross-Sectional Study on Their Occurrence and Associated Risk Factors with Environment and Public Health Importance. Zoonoses Public Health.

[14] Wang M., Wu S., Wang Y., Chen F., Shen Z., Lan Z. (2025). Antimicrobial Resistance Genes in Clinical *Escherichia coli* Strains from Livestock and Poultry in Shandong Province, China During 2015–2020. Antibiotics.

[15] Acharya J., Jha R., Gompo T.R., Chapagain S., Shrestha L., Rijal N., Shrestha A., Koirala P., Subedi S., Tamang B. (2024). Prevalence of Extended-Spectrum Beta-Lactamase (ESBL)-Producing *Escherichia coli* in Humans, Food, and Environment in Kathmandu, Nepal: Findings From ESBL *E. coli* Tricycle Project. Int. J. Microbiol..

[16] Widodo A., Khairullah A.R., Effendi M.H., Moses I.B., Agustin A.L.D. (2024). Extended-spectrum beta-lactamase-producing *Escherichia coli* from poultry: A review. Vet. World.

[17] Smet A., Martel A., Persoons D., Dewulf J., Heyndrickx M., Herman L., Haesebrouck F., Butaye P. (2009). Broad-spectrum β-lactamases among Enterobacteriaceae of animal origin: Molecular aspects, mobility and impact on public health. FEMS Microbiol. Rev..

[18] Effendi M., Witaningrum A. (2021). Cases of Multidrug Resistance (MDR) and Extended Spectrum Beta-Lactamase (ESBL) Producing *Escherichia Coli* from Broiler Chicken in Blitar, Indonesia. Biochem. Cell. Arch..

[19] Masruroh C.A., Sudarwanto M.B., Latif H. (2016). The Occurrence of extended spectrum B-Lactamase-producing *Escherichia coli* from broiler feces in Bogor. J. Sain Vet..

[20] Effendi M., Wibisono F., Witaningrum A., Permatasari D. (2021). Identification of Bla TEM and Bla SHV Genes of Extended Spectrum Beta Lactamase (ESBL) Producing *Escherichia coli* from Broilers Chicken in Blitar, Indonesia. Syst. Rev. Pharm..

[21] Reich F., Atanassova V., Klein G. (2013). Extended-spectrum β-lactamase- and AmpC-producing enterobacteria in healthy broiler chickens, Germany. Emerg. Infect. Dis..

[22] Ferreira M., Leao C., Clemente L., Albuquerque T., Amaro A. (2022). Antibiotic Susceptibility Profiles and Resistance Mechanisms to beta-Lactams and Polymyxins of *Escherichia coli* from Broilers Raised under Intensive and Extensive Production Systems. Microorganisms.

[23] WHO (2021). WHO Integrated Global Surveillance on ESBL-Producing E. coli Using a “One Health” Approach: Implementation and Opportunities.

[24] DGLAHS (2018). Survey Result: Antibiotic Use to Prevent Disease on Chicken Farms Is Still High.

[25] BPS (2022). Provinsi Kalimantan Barat Dalam Angka 2022.

[26] Siahaan S., Herman M.J., Fitri N. (2022). Antimicrobial Resistance Situation in Indonesia: A Challenge of Multisector and Global Coordination. J. Trop. Med..

[27] Hendriksen R.S., Cavaco L.M., Guerra B., Bortolaia V., Agersø Y., Svendsen C.A., Nielsen H.N., Kjeldgaard J.S., Pedersen S.K., Fertner M. (2023). Evaluation and validation of laboratory procedures for the surveillance of ESBL-, AmpC-, and carbapenemase-producing *Escherichia coli* from fresh meat and caecal samples. Front. Microbiol..

[28] Ministry of Agriculture (2022). Outlook Ayam Ras Pedaging 2022.

[29] Alonso C.A., Zarazaga M., Sallem R., Jouini A., Karim B.S., Torres C. (2017). Antibiotic resistance in *Escherichia coli* in husbandry animals: The African perspective. Lett. Appl. Microbiol..

[30] van den Bogaard A.E., London N., Driessen C., Stobberingh E.E. (2001). Antibiotic resistance of faecal *Escherichia coli* in poultry, poultry farmers and poultry slaughterers. J. Antimicrob. Chemother..

[31] Sáenz Y., Zarazaga M., Briñas L., Lantero M., Ruiz-Larrea F., Torres C. (2001). Antibiotic resistance in *Escherichia coli* isolates obtained from animals, foods and humans in Spain. Int. J. Antimicrob. Agents.

[32] Cantón R., Coque T.M. (2006). The CTX-M beta-lactamase pandemic. Curr. Opin. Microbiol..

[33] Moniri R.D.K. (2005). Fluoroquinolone-resistant *Escherichia coli* isolated from healthy broilers with previous exposure to fluoroquinolones. J. Infect. Dis. Antimicrob. Agents.

[34] Miranda J.M., Vázquez B.I., Fente C.A., Barros-Velázquez J., Cepeda A., Franco C.M. (2008). Evolution of resistance in poultry intestinal *Escherichia coli* during three commonly used antimicrobial therapeutic treatments in poultry. Poult. Sci..

[35] Horton R.A., Randall L., Snary E., Cockrem H., Lotz S., Wearing H., Duncan D., Rabie A., McLaren I., Watson E. (2011). Fecal Carriage and Shedding Density of CTX-M Extended-Spectrum β-Lactamase-Producing *Escherichia coli* in Cattle, Chickens, and Pigs: Implications for Environmental Contamination and Food Production. Appl. Environ. Microbiol..

[36] Ministry of Agriculture, Republic of Indonesia (2018). Annual Report.

[37] Wasnaeni Y., Iqbal A., Ismoyowati I. (2015). Broiler Farmers’ Behavior in Administering Antibiotic and Types of Antibiotic Content in Commercial Feed (A Case Study). Anim. Prod..

[38] Morosini M.I., García-Castillo M., Coque T.M., Valverde A., Novais A., Loza E., Baquero F., Cantón R. (2006). Antibiotic coresistance in extended-spectrum-beta-lactamase-producing Enterobacteriaceae and in vitro activity of tigecycline. Antimicrob. Agents Chemother..

[39] Yaghoubi S., Zekiy A.O., Krutova M., Gholami M., Kouhsari E., Sholeh M., Ghafouri Z., Maleki F. (2022). Tigecycline antibacterial activity, clinical effectiveness, and mechanisms and epidemiology of resistance: Narrative review. Eur. J. Clin. Microbiol. Infect. Dis..

[40] Basu S., Mukherjee M. (2018). Incidence and risk of co-transmission of plasmid-mediated quinolone resistance and extended-spectrum beta-lactamase genes in fluoroquinolone-resistant uropathogenic *Escherichia coli*: A first study from Kolkata, India. J. Glob. Antimicrob. Resist..

[41] Uddin T.M., Chakraborty A.J., Khusro A., Zidan B.R.M., Mitra S., Emran T.B., Dhama K., Ripon M.K.H., Gajdacs M., Sahibzada M.U.K. (2021). Antibiotic resistance in microbes: History, mechanisms, therapeutic strategies and future prospects. J. Infect. Public Health.

[42] Liu C., Wang P., Dai Y., Liu Y., Song Y., Yu L., Feng C., Liu M., Xie Z., Shang Y. (2021). Longitudinal monitoring of multidrug resistance in *Escherichia coli* on broiler chicken fattening farms in Shandong, China. Poult. Sci..

[43] Lukman D.W., Sudarwanto M.B., Purnawarman T., Latif H., Pisestyani H., Sukmawinata E., Akineden Ö. (2016). CTX-M-1 and CTX-M-55 producing *Escherichia coli* isolated from broiler feces in poultry slaughterhouse, Bogor, West Java Province. Glob. Adv. Res. J. Med. Med. Sci..

[44] Ejaz H., Younas S., Abosalif K.O.A., Junaid K., Alzahrani B., Alsrhani A., Abdalla A.E., Ullah M.I., Qamar M.U., Hamam S.S.M. (2021). Molecular analysis of blaSHV, blaTEM, and blaCTX-M in extended-spectrum β-lactamase producing Enterobacteriaceae recovered from fecal specimens of animals. PLoS ONE.

[45] Harijani N., Tyasningsih W., Effendi M. (2020). Biological Hazard on Multidrug Resistance (MDR) of *Escherichia Coli* Collected from Cloacal Swab of Broiler Chicken on Wet Markets Surabaya. Indian J. Forensic Med. Toxicol..

[46] Wang J., Stephan R., Karczmarczyk M., Yan Q., Hächler H., Fanning S. (2013). Molecular characterization of bla ESBL-harboring conjugative plasmids identified in multi-drug resistant *Escherichia coli* isolated from food-producing animals and healthy humans. Front. Microbiol..

[47] Che Y., Yang Y., Xu X., Břinda K., Polz M.F., Hanage W.P., Zhang T. (2021). Conjugative plasmids interact with insertion sequences to shape the horizontal transfer of antimicrobial resistance genes. Proc. Natl. Acad. Sci. USA.

[48] Brolund A., Rajer F., Giske C.G., Melefors Ö., Titelman E., Sandegren L. (2019). Dynamics of Resistance Plasmids in Extended-Spectrum-β-Lactamase-Producing Enterobacteriaceae during Postinfection Colonization. Antimicrob. Agents Chemother..

[49] Coura F., Diniz S., Silva M., Mussi J.M., Minharro S., Lage A., Heinemann M. (2015). Phylogenetic Group Determination of *Escherichia coli* Isolated from Animals Samples. Sci. World J..

[50] Teh H.B.H. (2018). Phylogeny and Virulence Factor Genes of Canine Urinary *Escherichia coli* in Relation to Clinical Disease and Antimicrobial Resistance. Master’s Thesis.

[51] Zhuge X., Zhou Z., Jiang M., Wang Z., Sun Y., Tang F., Xue F., Ren J., Dai J. (2021). Chicken-source *Escherichia coli* within phylogroup F shares virulence genotypes and is closely related to extraintestinal pathogenic *E. coli* causing human infections. Transbound Emerg. Dis..

[52] Özavci V., Yüksel T., Kirkan Ş. (2022). Phylogenetic characterization and determination of antibiotic susceptibility of avian pathogenic *Escherichia coli* strains isolated from broiler visceral organs. Rev. Científica Fac. Cienc. Vet..

[53] Wang M., Jiang M., Wang Z., Chen R., Zhuge X., Dai J. (2021). Characterization of Antimicrobial Resistance in Chicken-source Phylogroup F *Escherichia coli*: Similar Populations and Resistance Spectrums Between *E. coli* Recovered from Chicken Colibacillosis Tissues and Retail Raw Meats in Eastern China. Poult. Sci..

[54] CLSI (2021). Performance Standards for Antimicrobial Susceptibility Testing. Approved Standard. Informational Supplement.

[55] EUCAST Breakpoint Tables for Interpretation of MICs and Zone Diameters. http://www.eucast.org/clinical_breakpoints/.

[56] Schwarz S., Silley P., Simjee S., Woodford N., van Duijkeren E., Johnson A.P., Gaastra W. (2010). Assessing the antimicrobial susceptibility of bacteria obtained from animals. Vet. Microbiol..

[57] Hudzicki J. (2009). Kirby-Bauer disk diffusion susceptibility test protocol. Am. Soc. Microbiol..

[58] Perez F., Hujer A.M., Hujer K.M., Decker B.K., Rather P.N., Bonomo R.A. (2007). Global challenge of multidrug-resistant *Acinetobacter baumannii*. Antimicrob. Agents Chemother..

[59] Ogutu J., Zhang Q., Huang Y., Yan H., Su L., Gao B., Zhang W., Zhao J., Cai W., Li W. (2015). Development of a multiplex PCR system and its application in detection of blaSHV, blaTEM, blaCTX-M-1, blaCTX-M-9 and blaOXA-1 group genes in clinical *Klebsiella pneumoniae* and *Escherichia coli* strains. J. Antibiot..

[60] Peterson E., Kaur P. (2018). Antibiotic Resistance Mechanisms in Bacteria: Relationships Between Resistance Determinants of Antibiotic Producers, Environmental Bacteria, and Clinical Pathogens. Front. Microbiol..

[61] Batchelor M., Hopkins K., Threlfall E.J., Clifton-Hadley F.A., Stallwood A.D., Davies R.H., Liebana E. (2005). blaCTX-M Genes in Clinical *Salmonella* Isolates Recovered from Humans in England and Wales from 1992 to 2003. Antimicrob. Agents Chemother..

[62] Clermont O., Christenson J., Denamur E., Gordon D. (2013). The Clermont *Escherichia coli* phylo-typing method revisited: Improvement of specificity and detection of new phylo-groups. Environ. Microbiol. Rep..

