# Supplementary material for: Occurrence and Molecular Characterization of Extended-Spectrum Beta-Lactamase (ESBL)-Producing Escherichia coli in Broilers in Indonesia
Source: Antibiotics (Basel). 2025 Oct 15;14(10):1030. doi: 10.3390/antibiotics14101030 (PMC12561789; doi:10.3390/antibiotics14101030)
Supplement: Supplementary file 1 [file antibiotics-14-01030-s001.zip › antibiotics-3860692-supplementary.pdf]

Supplement table S1: Antimicrobial resistance patterns of ESBL-*E. coli*

No	Antimicrobial Resistance Pattern	n	Percentage (%)
1	CTX+AMP	2	1.0%
2	CTX+AMP+GEN	2	1.0%
3	NAL+COL+CAZ	1	0.5%
4	CAZ+CTX+AMP	1	0.5%
5	CAZ+CTX+AMP+GEN	1	0.5%
6	AZI+TET+CTX+AMP	1	0.5%
7	SMX+TMP+CTX+AMP	1	0.5%
8	NAL+TET+CTX+AMP+GEN	1	0.5%
9	CIP+NAL+CTX+AMP+GEN	1	0.5%
10	CIP+NAL+TET+CTX+AMP	1	0.5%
11	SMX+TET+CAZ+CTX+AMP	1	0.5%
12	SMX+CIP+NAL+CTX+AMP	1	0.5%
13	SMX+TMP+CTX+AMP+GEN	3	1.5%
14	SMX+TMP+TET+CTX+AMP	3	1.5%
15	CIP+NAL+CAZ+CTX+AMP+GEN	1	0.5%
16	CIP+NAL+CHL+TET+CTX+AMP	1	0.5%
17	TMP+COL+TET+CAZ+CTX+GEN	1	0.5%
18	TMP+CIP+NAL+CTX+AMP+GEN	1	0.5%
19	SMX+NAL+TET+CAZ+CTX+AMP	1	0.5%
20	SMX+CIP+NAL+TET+CTX+AMP	2	1.0%
21	SMX+CIP+NAL+COL+MER+CAZ	1	0.5%
22	SMX+TMP+CHL+CTX+AMP+GEN	1	0.5%
23	SMX+TMP+TET+CAZ+CTX+AMP	1	0.5%
24	SMX+TMP+AZI+CTX+AMP+GEN	8	4.0%
25	SMX+TMP+CIP+NAL+CTX+AMP	1	0.5%
26	NAL+COL+MER+TET+CAZ+CTX+GEN	1	0.5%
27	TMP+NAL+COL+TGC+CAZ+CTX+AMP	1	0.5%
28	TMP+CIP+NAL+CHL+TET+CTX+AMP	1	0.5%
29	SMX+NAL+TET+CAZ+CTX+AMP+GEN	1	0.5%
30	SMX+NAL+COL+TET+CAZ+CTX+AMP	1	0.5%
31	SMX+NAL+COL+AZI+TET+CAZ+CTX	1	0.5%
32	SMX+CIP+NAL+CAZ+CTX+AMP+GEN	2	1.0%
33	SMX+CIP+NAL+TET+CTX+AMP+GEN	2	1.0%
34	SMX+CIP+NAL+TET+CAZ+CTX+AMP	2	1.0%
35	SMX+CIP+NAL+AZI+CTX+AMP+GEN	1	0.5%
36	SMX+TMP+CHL+CAZ+CTX+AMP+GEN	1	0.5%
37	SMX+TMP+CHL+TET+CTX+AMP+GEN	1	0.5%
38	SMX+TMP+AZI+CAZ+CTX+AMP+GEN	7	3.5%
39	SMX+TMP+AZI+CHL+CTX+AMP+GEN	1	0.5%
40	SMX+TMP+AZI+TET+CTX+AMP+GEN	5	2.5%
41	SMX+TMP+MER+CAZ+CTX+AMP+GEN	1	0.5%
42	SMX+TMP+CIP+NAL+CTX+AMP+GEN	4	2.0%
43	SMX+TMP+CIP+NAL+CAZ+CTX+AMP	1	0.5%
44	SMX+TMP+CIP+NAL+TET+CTX+AMP	2	1.0%

No	Antimicrobial Resistance Pattern	n	Percentage (%)
45	TMP+CIP+NAL+AZI+TET+CTX+AMP+GEN	2	1.0%
46	SMX+CIP+NAL+CHL+CAZ+CTX+AMP+GEN	1	0.5%
47	SMX+TMP+AZI+CHL+CAZ+CTX+AMP+GEN	1	0.5%
48	SMX+TMP+AZI+TET+CAZ+CTX+AMP+GEN	2	1.0%
49	SMX+TMP+COL+TET+CAZ+CTX+AMP+GEN	1	0.5%
50	SMX+TMP+CIP+AZI+CAZ+CTX+AMP+GEN	8	4.0%
51	SMX+TMP+CIP+AZI+TET+CTX+AMP+GEN	1	0.5%
52	SMX+TMP+CIP+NAL+CAZ+CTX+AMP+GEN	2	1.0%
53	SMX+TMP+CIP+NAL+TET+CTX+AMP+GEN	7	3.5%
54	SMX+TMP+CIP+NAL+AZI+CTX+AMP+GEN	8	4.0%
55	SMX+TMP+CIP+NAL+COL+AZI+CTX+AMP	1	0.5%
56	TMP+CIP+NAL+AZI+CHL+CAZ+CTX+AMP+GEN	1	0.5%
57	TMP+CIP+NAL+AZI+TET+CAZ+CTX+AMP+GEN	4	2.0%
58	SMX+TMP+COL+AZI+TET+CAZ+CTX+AMP+GEN	1	0.5%
59	SMX+TMP+NAL+COL+CHL+TET+CAZ+CTX+AMP	1	0.5%
60	SMX+TMP+CIP+AZI+TET+CAZ+CTX+AMP+GEN	1	0.5%
61	SMX+TMP+CIP+NAL+CHL+CAZ+CTX+AMP+GEN	1	0.5%
62	SMX+TMP+CIP+NAL+CHL+TET+CTX+AMP+GEN	3	1.5%
63	SMX+TMP+CIP+NAL+CHL+TET+CAZ+CTX+AMP	1	0.5%
64	SMX+TMP+CIP+NAL+AZI+CAZ+CTX+AMP+GEN	15	7.5%
65	SMX+TMP+CIP+NAL+AZI+TET+CTX+AMP+GEN	16	8.0%
66	SMX+TMP+CIP+NAL+COL+CAZ+CTX+AMP+GEN	1	0.5%
67	SMX+TMP+CIP+NAL+COL+TET+CTX+AMP+GEN	3	1.5%
68	SMX+TMP+CIP+NAL+COL+AZI+CTX+AMP+GEN	1	0.5%
69	SMX+TMP+CIP+NAL+AZI++CHL+CTX+AMP+GEN	2	1.0%
70	TMP+CIP+NAL+AZI+CHL+TET+CAZ+CTX+AMP+GEN	1	0.5%
71	TMP+CIP+NAL+COL+AZI+CHL+TET+CTX+AMP+GEN	1	0.5%
72	SMX+CIP+NAL+COL+MER+TET+CAZ+CTX+AMP+GEN	1	0.5%
73	SMX+TMP+CIP+NAL+AZI+TET+CAZ+CTX+AMP+GEN	15	7.5%
74	SMX+TMP+CIP+NAL+AZI+CHL+TET+CTX+AMP+GEN	3	1.5%
75	SMX+TMP+CIP+NAL+COL+CHL+TET+TGC+CTX+AMP	1	0.5%
76	SMX+TMP+CIP+NAL+COL+AZI+CAZ+CTX+AMP+GEN	3	1.5%
77	SMX+TMP+CIP+NAL+COL+AZI+TET+CTX+AMP+GEN	1	0.5%
78	SMX+TMP+CIP+NAL+AZI+CHL+TET+CAZ+CTX+AMP+GEN	6	3.0%
79	SMX+TMP+CIP+NAL+COL+CHL+TET+CAZ+CTX+AMP+GEN	1	0.5%
80	SMX+TMP+CIP+NAL+COL+AZI+CHL+TET+CTX+AMP+GEN	1	0.5%
81	SMX+TMP+CIP+NAL+COL+MER+AZI+TET+CAZ+AMP+GEN	1	0.5%
82	SMX+TMP+CIP+NAL+AZI+CHL+TET+TGC+CAZ+CTX+AMP+GEN	1	0.5%
83	SMX+TMP+CIP+NAL+COL+AZI+TET++TGC+CAZ+CTX+AMP+GEN	1	0.5%
84	SMX+TMP+CIP+NAL+COL++AZI+CHL+TET+CAZ+CTX+AMP+GEN	2	1.0%
85	SMX+TMP+CIP+NAL+MER+AZI+CHL+TET+TGC+CAZ+CTX+AMP+GE	1	0.5%
		200	100%
